# Case Report: response of HER2-positive ductal carcinoma *in situ* to osimertinib with supporting *in vitro* evidence

**DOI:** 10.3389/fonc.2026.1845658

**Published:** 2026-05-20

**Authors:** Shogo Baba, Mami Koketsu, Hajime Kuroda, Megumi Suzuki, Hiroshi Nishihara, Yasutaka Kato, Hiroyuki Kawami, Oi Harada

**Affiliations:** 1 Department of Pathology and Genetics, Laboratory of Cancer Medical Science, Hokuto Hospital, Obihiro, Hokkaido, Japan; 2 Department of Diagnostic Pathology, Adachi Medical Center, Tokyo Women’s Medical University, Adachi, Tokyo, Japan; 3 Department of Clinical Pathology, Hokuto Hospital, Obihiro, Hokkaido, Japan; 4 Megumi Breast Clinic, Obihiro, Hokkaido, Japan; 5 Center for Cancer Genomics, Keio University School of Medicine, Shinjuku, Tokyo, Japan; 6 Center for Breast Diseases and Breast Cancer, Hokuto Hospital, Obihiro, Hokkaido, Japan; 7 Department of Clinical Pathology, Kameda Medical Center Laboratory, Kamogawa, Chiba, Japan

**Keywords:** breast cancer, DCIS, EGFR, HER2, osimertinib

## Abstract

Ductal carcinoma *in situ* (DCIS) carries a risk of progression to invasive ductal carcinoma (IDC), and local treatments such as mastectomy and radiation therapy are commonly used. This case report describes a 74-year-old Japanese woman with concurrent HER2-positive DCIS and non-small cell lung cancer (NSCLC) with EGFR mutation who showed a remarkable response to osimertinib, an epidermal growth factor receptor inhibitor used to treat NSCLC. At initial presentation, the breast lesion measured 28 × 11 × 29 mm. Biopsy revealed high-grade DCIS with a HER2-immunohistochemistry score of 3+ and a Ki-67 index of 20–30%. The NSCLC was subsequently resected, and adjuvant osimertinib therapy was initiated postoperatively. Two months after treatment initiation, a right mastectomy was performed. Postoperative pathological examination showed a marked reduction in tumor size to 8 × 3 mm and a marked decrease in Ki-67 to 1%. These findings indicated multiple post-chemotherapy changes, with only a small amount of high-grade DCIS remaining, suggesting a therapeutic effect of the lung cancer treatment. Assessment of the inhibitory effect of osimertinib on HER2 *in vitro* demonstrated that osimertinib inhibited cell proliferation in a HER2 expression-dependent way. Western blotting also suggested the inhibition of HER2 phosphorylation. Therefore, these findings suggest that the remarkable response of HER2-positive DCIS in this case may be attributable to the HER2-inhibitory effect of osimertinib. Further research is warranted to determine whether osimertinib could serve as a potential treatment option for HER2-positive breast cancer, including DCIS.

## 1 Introduction

Ductal carcinoma *in situ* (DCIS) is the most common type of non-invasive breast cancer, accounting for approximately 15–25% of all breast cancers detected through screening (1, 2). DCIS is characterized by abnormal epithelial cells confined to the milk ducts of the breast and is considered a precursor of invasive ductal carcinoma (IDC). Although DCIS remains confined to the ducts, it poses a risk of progression to IDC; therefore, local therapies such as mastectomy and radiation therapy are generally recommended (3). Determining the hormone receptor status and Ki-67 proliferation index is important for the pathological diagnosis of breast cancer, including DCIS. HER2 -positive DCIS is more frequently observed than in IDC and is associated with high-grade histology and an increased risk of local recurrence. Nevertheless, the standard treatment for HER2-positive DCIS remains the same as for other DCIS subtypes, consisting of surgical resection with or without adjuvant radiotherapy (4, 5). Evidence regarding the therapeutic efficacy of anti-HER2 agents, such as trastuzumab, remains limited, and HER2 status is not currently used as a basis for treatment decision-making (6).

Osimertinib is an oral third-generation epidermal growth factor receptor (EGFR) tyrosine kinase inhibitor (TKI) (7–9). Preclinical studies demonstrated that osimertinib potently inhibited phosphorylation and suppressed cell proliferation in cell lines harboring the EGFR T790M resistance mutation as well as EGFR-sensitizing, with high selectivity for mutant EGFR and minimal off-target activity across approximately 280 kinases (8). Notably, these studies also suggested potential inhibitory activity against HER2 and HER4 kinases. Currently, osimertinib is widely used for the treatment of advanced non-small cell lung cancer (NSCLC) harboring EGFR mutations.

In this study, we report a remarkable response in a patient with HER2-positive DCIS treated with osimertinib, which was used to treat concurrent NSCLC, incorporating *in vitro* experiments.

## 2 Case description

**Figure 1:**
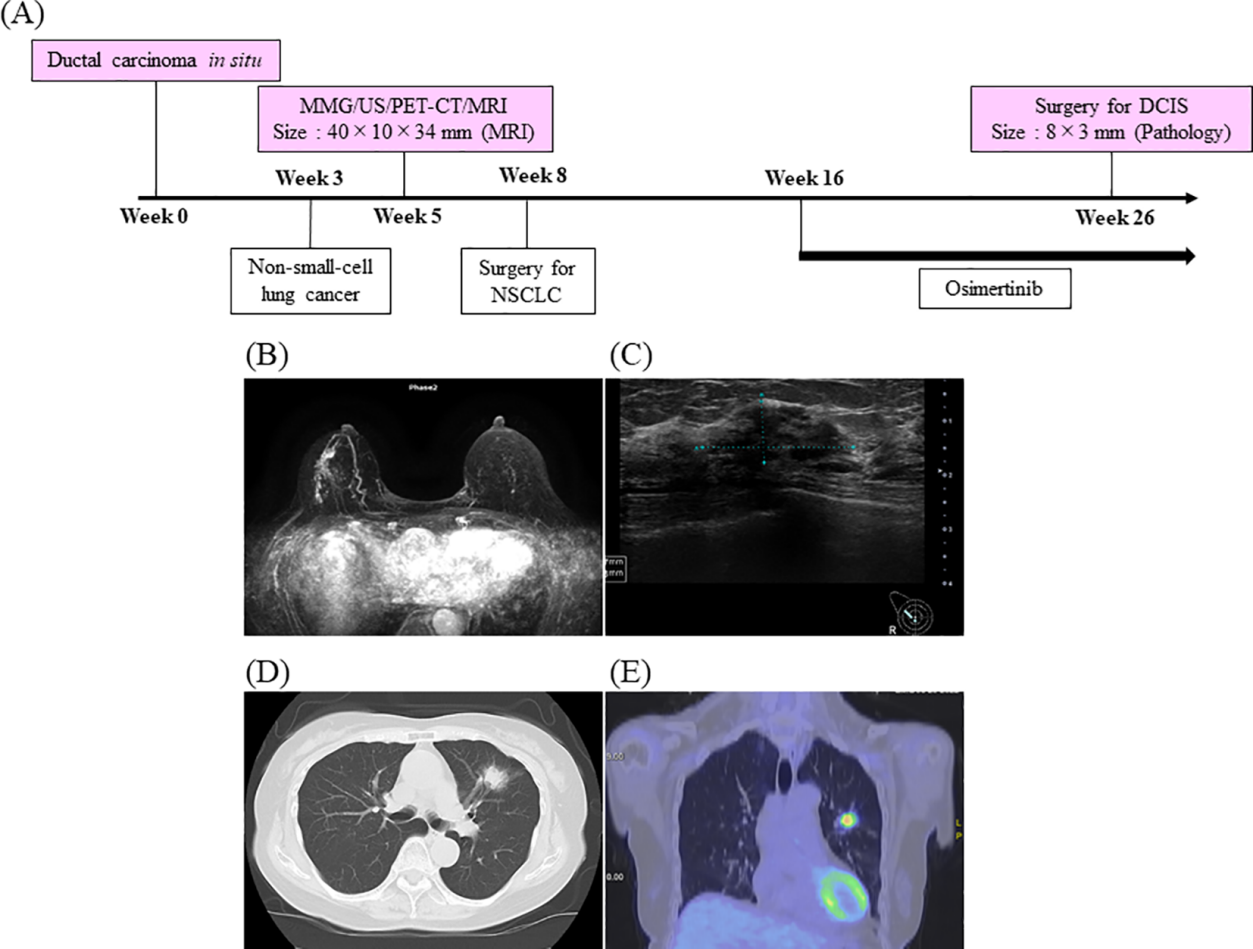
Timeline and diagnostic images. Timeline of the diagnosis and treatment process for the patient **(A)** and pretreatment diagnostic images of the breast and lung **(B–E)**. Mammography reveals focal microcalcifications and sub-nipple calcifications in the upper outer quadrant of the right breast **(B)**, whereas ultrasound reveals an irregular hypoechoic area measuring 28 × 11 × 29 mm **(C)**. CT reveals a 2.5-cm tumor shadow with irregular margins in the left upper lobe S3a **(D)**, and positron emission tomography-computed tomography demonstrates uptake in the right breast and left upper lobe **(E)**.

**Figure 2:**
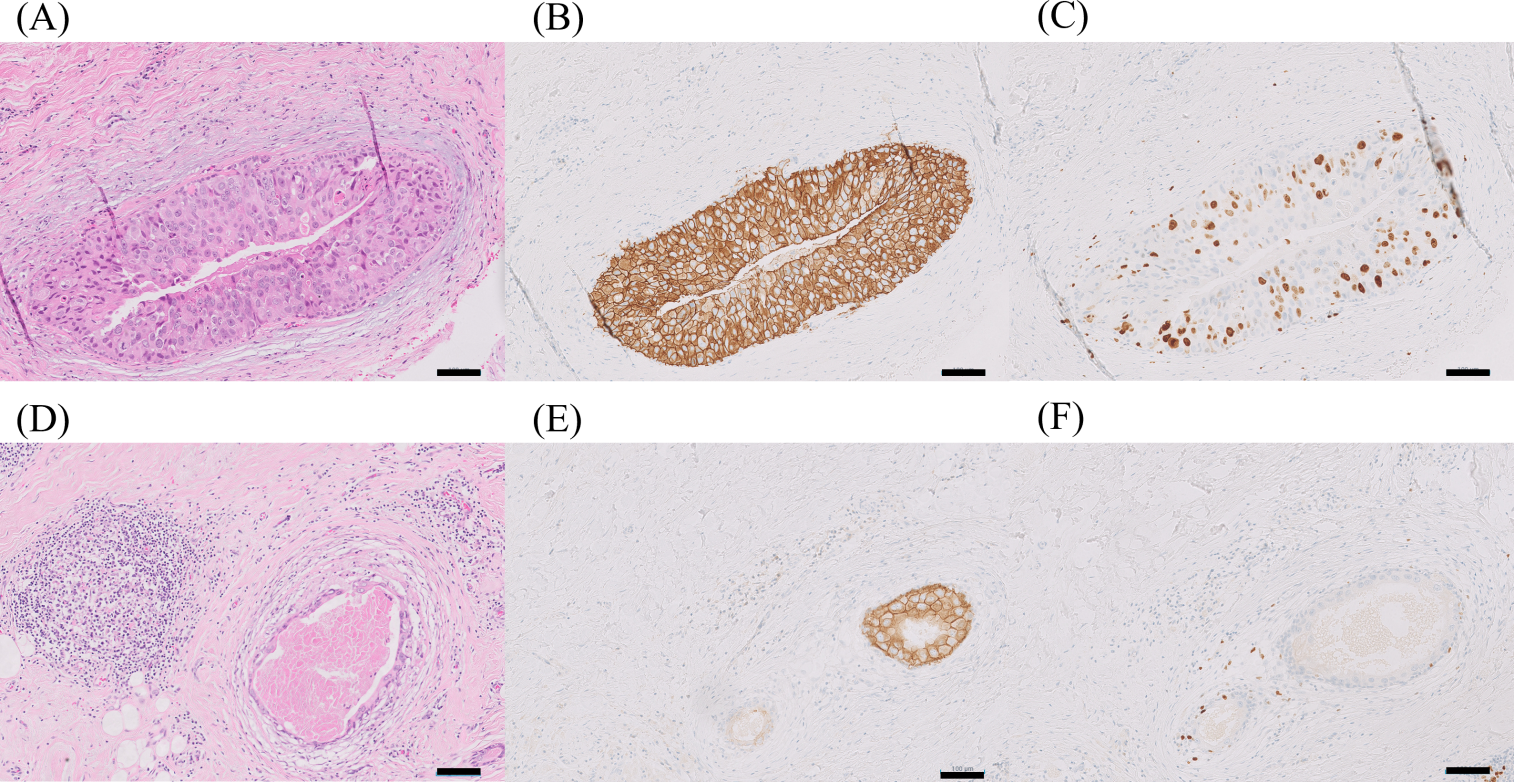
Comparison of HE, HER2-IHC, and Ki-67 in biopsy and surgical specimens. Hematoxylin and eosin (HE; **A, D**), HER2-IHC **(B, E)**, and Ki-67 **(C, F)** staining of biopsy specimens before osimertinib treatment **(A–C)** and surgical specimens after treatment **(D–F)**. In the surgical specimens, foamy cell clusters with foreign body reactions, inflammatory cell infiltration, hemosiderin deposition, and lymphoid follicle formation are observed around DCIS. The Ki-67 level is 1%, which shows a marked decrease. The high-grade DCIS observed in the biopsy specimen is present in very small amounts **(D–F)** (scale bar=100 µm).

A 74-year-old Japanese woman was diagnosed with DCIS based on a needle biopsy performed at another hospital (Week 0) (**Figure 1A**). Histologically, the lesion was classified as high-grade DCIS according to the WHO nuclear grading system. Immunohistochemistry (IHC) revealed hormone receptor negativity, a HER2-IHC score of 3+, and a Ki-67 index of 20–30% (**Figures 2A–C**). She had a history of dyslipidemia but no other significant comorbidities or medication use. Her family history included colorectal cancer in her mother and breast cancer in her niece. Custom gene panel testing identified actionable alterations, including ERBB2 amplification (Copy number; CN = 7.75), IDH2 amplification (CN = 5.86), and VHL loss (CN = 0.43), with no EGFR mutations or amplifications detected.

During the same period, a 2.5-cm irregularly margined mass was detected in segment S3a of the left upper lobe of her lung. At Week 3, a transbronchial biopsy confirmed the diagnosis of lung adenocarcinoma (**Figures 1A, D**).

The patient was referred to our institution for surgical management of DCIS at Week 5. Breast ultrasonography and mammography revealed focal and subpapillary microcalcifications in the upper outer quadrant of the right breast, along with a 28 × 11 × 29-mm heterogeneous hypoechoic area (**Figures 1B, C**). Breast MRI demonstrated focal non-mass enhancement in the right C region, measuring 40 × 10 × 34 mm, with a fast plateau enhancement pattern on T1-weighted imaging. Given the extensive lesion distribution, mastectomy was scheduled. Positron emission tomography (PET) imaging showed abnormal uptake in both the right breast and left upper lobe of the lung, and surgical treatment of lung cancer was prioritized (**Figure 1E**).

At Week 8, left upper lobectomy was performed at another institution. Histopathological examination revealed pT2a (3.6 cm), N2 (5/11), and M0 disease, corresponding to pathological stage IIIA. Because the tumor harbored an EGFR p.L858R mutation, adjuvant oral osimertinib therapy was initiated at Week 16, with a planned duration of 3 years.

At Week 26, right mastectomy and sentinel lymph node biopsy (SNB) were performed. No lymph node metastases were detected. Postoperative pathological examination revealed DCIS measuring 8 × 3 mm, with an intermediate WHO nuclear grade, hormone receptor negativity, and a HER2-IHC score of 3 +. The Ki-67 index was 1%, showing a marked decrease compared with that of the initial biopsy sample.

## 3 *In vitro* HER2 inhibition assay with osimertinib

### 3.1 HER2 expression analysis in breast cancer cell lines

To confirm the efficacy of osimertinib against breast cancer in this context, experiments were conducted using six breast cancer cell lines: MCF7, T47D, ZR-75-1, MDA-MB-453, SK-BR-3, and BT474. Detailed experimental procedures are described in Supplementary Materials. Initially, quantitative reverse transcription polymerase chain reaction and Western blotting were used to assess HER2 mRNA and protein expression (see Supplementary Materials). HER2 mRNA expression levels were low in MCF7, T47D, and ZR-75–1 cells, moderate in MDA-MB-453 cells, and high in BT474 and SK-BR-3 cells (**Supplementary Figure 1A**). Protein expression levels were generally consistent with mRNA expression levels, except in ZR-75–1 cells, which showed intermediate HER2 protein expression (**Supplementary Figure 1B**).

### 3.2 Gene panel analysis of breast cancer cell lines

Gene panel testing was performed as described in Supplementary Materials. EGFR mutations did not detect in any cell lines (**Supplementary Table 1**). ERBB2 amplification was detected in MDA-MB-453 (CN = 4.74), BT474 (CN = 27.57), and SK-BR-3 (CN = 19.82).

### 3.3 Evaluation of osimertinib sensitivity using cell proliferation assays

**Figure 3:**
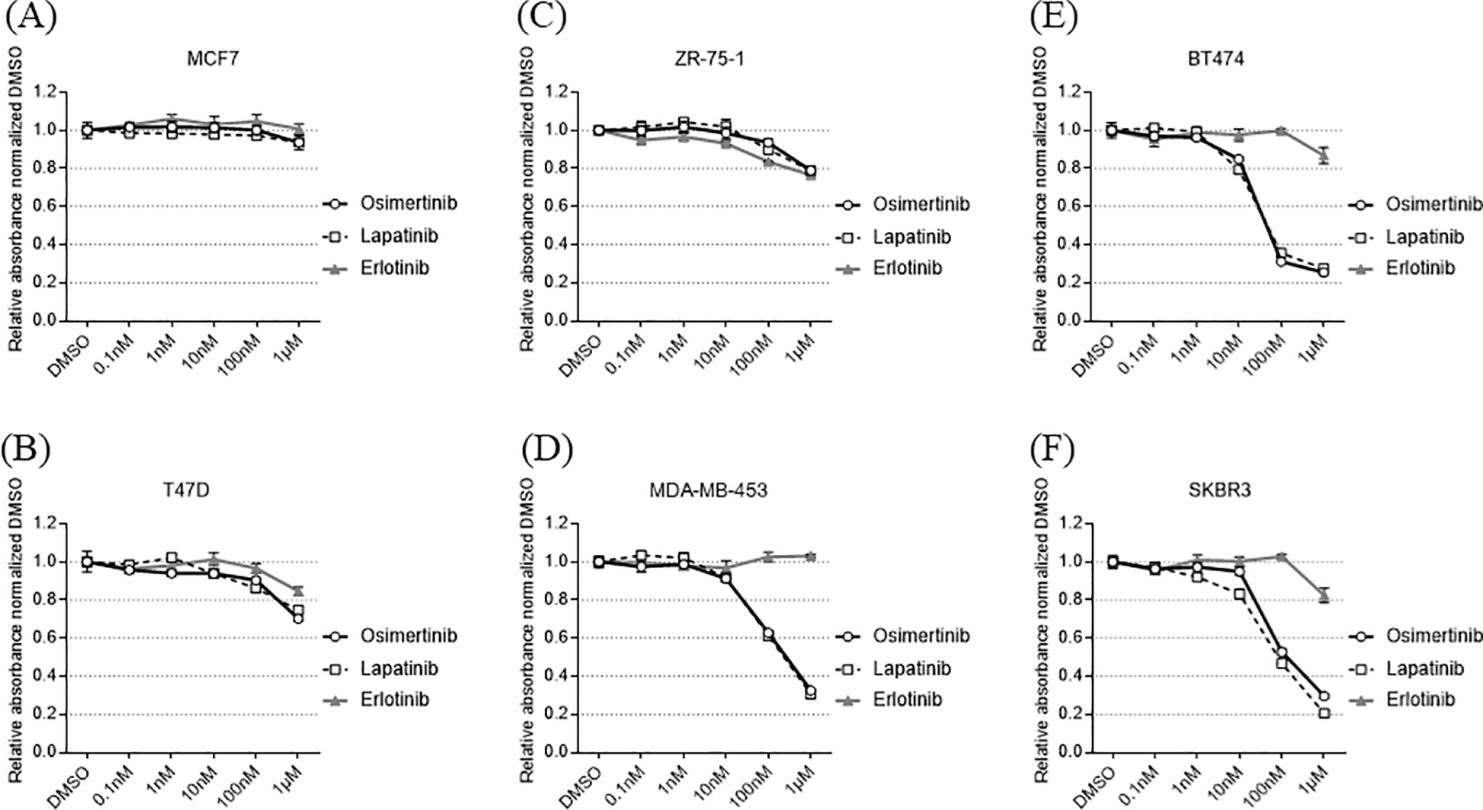
Cell proliferation assay using cell lines. Osimertinib sensitivity is evaluated using a cell proliferation assay. Osimertinib shows no strong proliferation inhibitory effect in HER2-low-expressing cell lines **(A, B)**, whereas it exhibits proliferation inhibition comparable to that of lapatinib in cell lines expressing high levels of HER2 **(E, F)**. Among cell lines expressing moderate levels of HER2 (ZR-75-1) there is minimal effect **(C)**, whereas MDA-MB-453 shows a significant effect **(D)**.

Osimertinib sensitivity was evaluated using a cell proliferation assay as described in **Supplementary Materials**. Osimertinib did not exhibit strong anti-proliferation effects in HER2-low-expression cell lines, whereas it demonstrated anti-proliferation effects comparable to those of lapatinib in HER2-high-expression cell lines (**Figure 3**). Among cell lines with intermediate HER2 protein expression, MDA-MB-453—which exhibited ERBB2 gene amplification—showed an anti-proliferation effect, whereas ZR-75-1—which lacked gene amplification—showed a minimal effect.

### 3.4 Effects of osimertinib on HER2 phosphorylation

**Figure 4:**
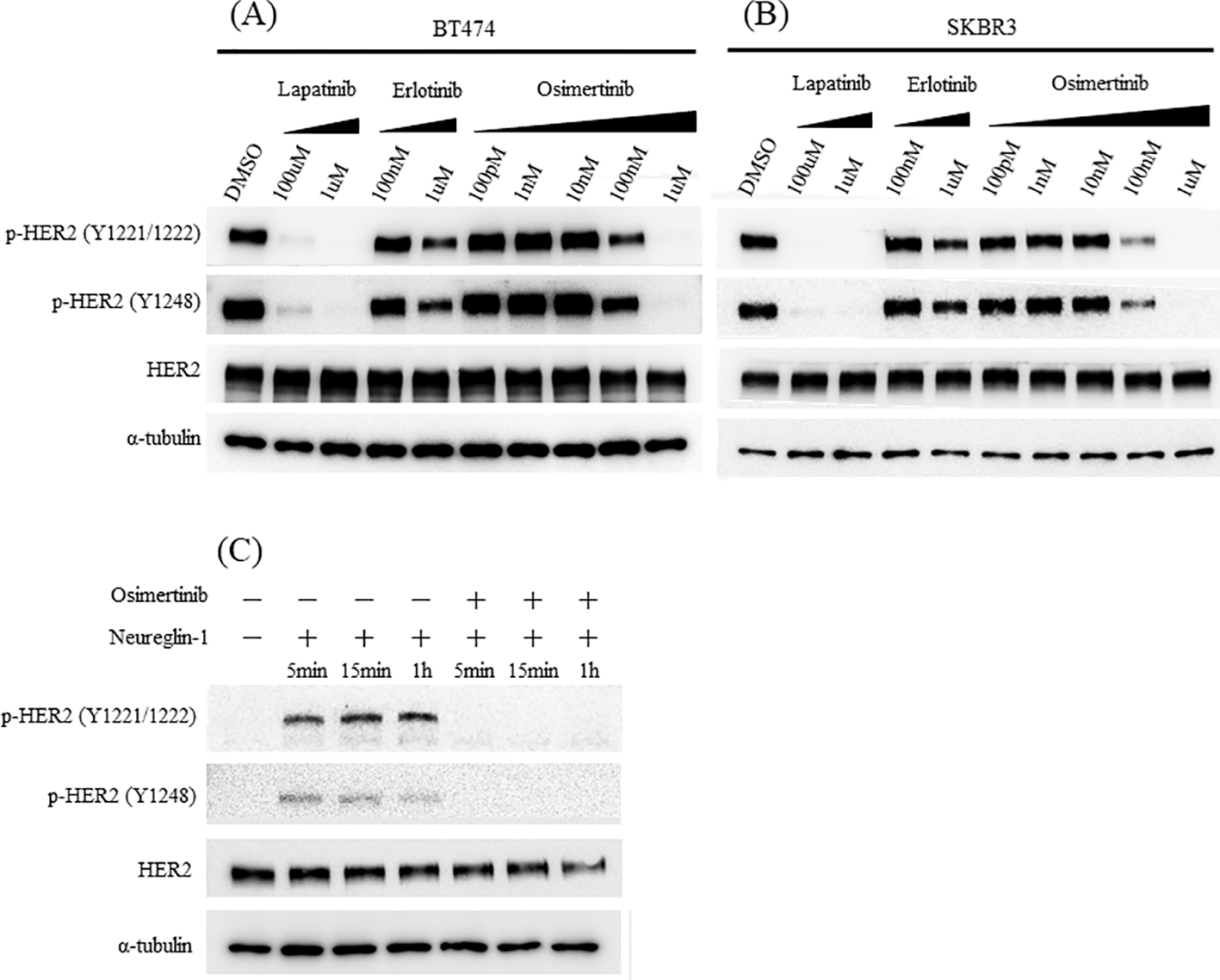
HER2 phosphorylation inhibition assay. In cell lines expressing high levels of HER2 (SKBR3 and BT474), osimertinib inhibits HER2 phosphorylation in a concentration-dependent manner **(A, B)**. In the MCF7 cell line which expresses low levels of HER2, osimertinib inhibits HER2 phosphorylation induced by Neureglin1 **(C)**.

To determine whether osimertinib directly targets the HER2 protein, we examined its effect on HER2 phosphorylation as described in **Supplementary Materials** in cell lines with high HER2 expression. A decrease in phosphorylated HER2 expression started 1 hour after osimertinib addition (**Supplementary Figure 2**), indicating a concentration-dependent inhibition (**Figures 4A, B**).

### 3.5 Effects of osimertinib on ligand-dependent HER2 phosphorylation

We investigated whether osimertinib inhibits ligand-dependent HER2 phosphorylation. Neureglin1—a ligand for HER3 and HER4 that promotes HER2 heterodimerization and phosphorylation—was added to the low HER2 expression cell line, MCF7. Osimertinib inhibited Neureglin1-mediated HER2 phosphorylation in these cells (**Figure 4C**).

## 4 Discussion

Osimertinib is a third-generation EGFR-TKI used for the treatment of NSCLC with EGFR mutations. It selectively inhibits both EGFR-TKI-sensitive mutations and the EGFR p.T790M resistance mutation (9). Regarding its target selectivity, screening against a panel of 280 kinases identified only a limited number of potentially inhibitable kinases, including HER2, HER4, and ACK1 (8). However, apart from EGFR, none of these kinases are recognized as therapeutic targets for osimertinib, and their clinical relevance remains unclear.

We recently encountered a case of HER2-positive DCIS in which osimertinib, administered as adjuvant therapy for NSCLC, appeared to produce a marked therapeutic response. The potential efficacy of osimertinib against HER2-positive tumors has been previously reported in lung cancer, where potent antitumor effects were observed in mouse models of HER2-overexpressing lung cancer (10). However, to the best of our knowledge, its efficacy has not been evaluated in invasive types of breast cancer, let alone non-invasive types such as DCIS. In our *in vitro* experiments, osimertinib demonstrated HER2 expression-dependent growth inhibition in breast cancer cell lines lacking EGFR mutations. Furthermore, similar to lapatinib, osimertinib inhibited HER2 phosphorylation, suggesting a potential anti-tumor effect mediated through targeting HER2 rather than EGFR. Additionally, osimertinib appeared to inhibit ligand-dependent HER2 phosphorylation through heterodimer formation with HER3 and other receptor proteins. However, the observed decrease in HER2 phosphorylation may also reflect indirect effects on HER2-related signaling, possibly mediated through inhibition of downstream signaling pathways. Furthermore, although validation using primary cells derived from the patient’s biopsy specimens would have been valuable, no viable specimens remained for additional *in vitro* analyses. Therefore, we were unable to perform these experiments.

For HER2-positive advanced or metastatic recurrent breast cancer, the combination of lapatinib and chemotherapy is approved as an EGFR/HER2-TKI-based therapy. In contrast, osimertinib may be effective as a single agent, and could also be effective for DCIS, for which limited treatment options are available. Although side effects such as interstitial pneumonia, myelosuppression, and hepatic dysfunction have been reported, osimertinib is relatively better tolerated compared with conventional EGFR-TKIs (7). Therefore, it may warrant consideration as a potential therapeutic option in breast cancer.

In summary, although a causal relationship cannot be definitively established from a single case, this case report demonstrates the potential efficacy of osimertinib against HER2-positive DCIS in a real-world clinical setting. Importantly, this is one of the few studies to examine the potential of osimertinib as an anti-HER2 agent in breast cancer treatment. Future research may clarify whether osimertinib could expand treatment options for HER2-positive breast cancer, including DCIS.

## Data Availability

The data analyzed in this study is subject to the following licenses/restrictions: Data supporting the results of this study are not publicly available due to patient confidentiality and ethical restrictions imposed by the Ethics Committee of Hokuto Hospital. Requests to access these datasets should be directed to the corresponding author, subject to institutional and ethical approvals.
