# Supplementary material for: Case Report: response of HER2-positive ductal carcinoma in situ to osimertinib with supporting in vitro evidence
Source: Front Oncol. 2026 May 20;16:1845658. doi: 10.3389/fonc.2026.1845658 (PMC13229710; doi:10.3389/fonc.2026.1845658)
Supplement: Supplementary file 1 [file DataSheet1.docx]

Supplementary Material

Case Report: Remarkable Response of HER2-Positive Ductal Carcinoma *in Situ* to Osimertinib with Supporting *In Vitro* Evidence

Shogo Baba^1^, Mami Koketsu^1^, Hajime Kuroda^2^, Megumi Suzuki^3^, Hiroshi Nishihara^1,4^, Yasutaka Kato^1,4^, Hiroyuki Kawami^1,5^, Oi Harada^6^

*** Correspondence:** Shogo Baba: s-baba@hokuto7.or.jp

**1 Supplementary Materials and Methods**

**1.1 Cell culture**

Two human breast cancer cell lines, MCF7 and SK-BR-3, were obtained from the American Type Culture Collection (ATCC) and were kindly provided by Dr. Ujiie (Asahikawa Medical University, Asahikawa, Japan). Four additional ATCC-derived cell lines (T-47D, ZR-75-1, MDA-MB-453, and BT-474) were purchased from Keio University and utilised through a collaborative research agreement. All cell lines were routinely screened and confirmed to be negative for mycoplasma contamination. The cells were maintained according to the manufacturer’s protocols. MDA-MB-453 cells were cultured at 37°C in a humidified incubator under a CO_2_-free atmosphere, while all other cell lines were maintained with 5% CO_2_ supplementation.

**1.2 Gene panel testing (GPT)**

Genetic analyses were performed according to previously established procedures. Total DNA was extracted from cultured cells using a DNeasy Blood & Tissue Kit (QIAGEN, Hilden, Germany). Libraries were prepared using SureSelect XT Low Input Reagents with SureSelect PrePool Custom Tier2 (143-gene panel) (Agilent, Santa Clara, CA, USA). Next-generation sequencing (NGS) was conducted using a MiSeq instrument (Illumina, San Diego, CA, USA). Gene variant analyses were performed on the resulting FASTQ files using the Genome Jack analysis pipeline (Mitsubishi Electric Software; <https://genomejack.net/>).

**1.3 Gene expression assay by quantitative RT-qPCR**

Total RNA was isolated from cell cultures using TRIzol reagent (Thermo Fisher Scientific, Waltham, MA, USA) following the manufacturer’s instructions. RNA concentration was quantified using a Qubit fluorometer (Thermo Fisher Scientific), and first-strand cDNA synthesis was performed using the Transcriptor Universal cDNA Master kit (Merck KGaA, Darmstadt, Germany). Quantitative RT-PCR (qRT-PCR) was conducted in triplicate for each gene on a QuantStudio 5 Real-Time PCR System (Thermo Fisher Scientific) using PowerUP SYBR Green Master Mix (Thermo Fisher Scientific). The primer sequences used were as follows:

- *HER2*-F: 5’-GGAAGTACACGATGCGGAGACT-3’
- *HER2*-R: 5’-ACCTTCCTCAGCTCCGTCTCTT-3’
- *ACTB*-F: 5’-GCCTCGCCTTTGCCGATCC-3’
- *ACTB*-R: 5’-GCGCGGCGATATCATCATCCA-3’

Relative gene expression was calculated using the 2^-ΔΔ^*^Ct^* method with *ACTB* as the internal reference gene.

**1.4 Cell proliferation assay**

Cells (1 × 10^3^) were plated into each well of a 96-well plate, and drugs (osimertinib, lapatinib, and erlotinib) were added the next day. Cell proliferation assays were performed in triplicate for each condition. Three days after drug addition, the CellTiter 96 AQueous One Solution Cell Proliferation Assay System (Promega, Madison, WI, USA) was used according to the manufacturer’s instructions. After 2 h of incubation in a humidified atmosphere, the absorbance at 490 nm was measured using a Multiskan FC Microplate Photometer (Thermo Fisher Scientific).

**1.5 Western blotting**

Cells were harvested and lysed using the EzRIPA Lysis Kit (ATTO, Tokyo, Japan) according to the manufacturer’s guidelines. Protein concentrations were determined using a Qubit fluorometer (Thermo Fisher Scientific). Western blotting was performed to analyse HER2, p-HER2 (Y1221/1222), p-HER2 (Y1248), and α-tubulin levels in MCF7, SK-BR-3, and BT-474 cell lines. Briefly, 50 μg of total protein per sample was resolved via SDS-PAGE and transferred to a PVDF membrane (Merck KGaA). After blocking with nonfat dry milk (Cell Signaling Technology, Danvers, MA, USA), the membranes were incubated with primary antibodies against HER2, p-HER2 (Y1221/1222), p-HER2 (Y1248), and α-tubulin overnight at 4°C.

Following primary incubation, the membranes were washed and incubated with HRP-conjugated secondary antibodies for 1 h at room temperature. Signals were detected using Immobilon Western Chemiluminescent HRP Substrate (Merck KGaA). The antibodies used included HER2/ErbB2 (D8F12) XP Rabbit mAb #4290, phospho-HER2/ErbB2 (Tyr1221/1222) (6B12) rabbit mAb #2243, phospho-HER2/ErbB2 (Tyr1248) antibody #2247, anti-rabbit IgG, HRP-linked antibody #7074 (Cell Signalling Technology), anti-α-tubulin mouse mAb (formerly Oncogene Research Products, Cambridge, MA, USA), and goat anti-mouse immunoglobulin/HRP P0447 (Agilent).

**2 Supplementary Figures and Tables**

**2.1 Supplementary Figures**





**Supplementary Figure 1. HER2 mRNA and protein expression in breast cancer cell lines.** HER2 mRNA and protein expression are confirmed using quantitative reverse transcription-polymerase chain reaction **(A)** and Western blotting **(B)**. Both mRNA and protein expression analyses show that MCF7 and T47D cells expressed low levels of HER2, whereas BT474 and SK-BR-3 cells expressed high levels. In ZR-75-1 cells, mRNA expression is low, whereas protein expression is moderate and similar to that observed in MDA-MB-453 cells.





**Supplementary Figure 2. Time-dependent inhibition assay of HER2 phosphorylation.** Time-course changes in phosphorylated HER2 in the HER2-high-expressing cell line, SKBR3, upon addition of osimertinib (1 μg/ml). Phosphorylated HER2 expression decreased after 1 h.

**2.2 Supplementary Table**

**Supplementary Table 1. Gene panel test results in the cell lines.** The gene panel test did not detect any epidermal growth factor receptor mutations in any of the cell lines. ERBB2 amplification was detected in MBA-MB-453 (CN=4.74), BT474 (CN=27.57), and SK-BR-3 (CN=19.82).

|  | **SNV** | **Amplification** | **Loss** |
| --- | --- | --- | --- |
| **MCF7** | *PIK3CA* | *NRAS* | *CDKN2A* |
| **(Luminal A)** |  | *MYC* | *SMAD4* |
|  |  | *GNAS* | *KDM6A* |
|  |  |  | *ATRX* |
|  |  |  | *ATM* |
| **T47D** | *PIK3CA* | *PIK3CA* | *CDKN2A* |
| **(Luminal A/B)** | *ARID1A* | *PRKCI* |  |
|  | *TP53* |  |  |
| **ZR-75-1** | *JAK3* | *CCND1* | *CDKN2A* |
| **(Luminal A)** | *PTEN* | *MDM4* | *ENO1* |
|  |  | *FGFR1* | *STK11* |
|  |  | *CDK4* | *SMARCA4* |
|  |  | *MDM2* |  |
| **MDA-MB-453** | *PIK3CA* | *ERBB2* (CN=4.74) | *RAD51C* |
| **(TNBC)** | *FGFR4* | *CCND1* | *CDKN2A* |
|  | *CDH1* | *MDM4* | *TP53* |
|  |  |  | *PTCH1* |
| **BT474** | *PIK3CA* | *ERBB2* (CN=27.57) | *ATRX* |
| **(Lumina-HER2)** | *TP53* |  | *CDKN2A* |
|  | *BRCA2* |  |  |
| **SK-BR-3** | *TP53* | *ERBB2* (CN=19.82) | *BAP1* |
| **(pure-HER2)** |  | *MYC* | *PBRM1* |
|  |  | *STAT3* |  |
|  |  | *SRC* |  |
|  |  | *RAC1* |  |
|  |  | *EGFR* |  |
|  |  | *MET* |  |
|  |  | *GNAS* |  |
